# Metabolite Support of Long-Term Storage of Sperm in the Spermatheca of Honeybee (*Apis mellifera*) Queens

**DOI:** 10.3389/fphys.2020.574856

**Published:** 2020-11-10

**Authors:** Zhenguo Liu, Feng Liu, Guilin Li, Xuepeng Chi, Ying Wang, Hongfang Wang, Lanting Ma, Kai Han, Guangdong Zhao, Xingqi Guo, Baohua Xu

**Affiliations:** ^1^College of Animal Science and Technology, Shandong Agricultural University, Tai’an, China; ^2^Apiculture Institute of Jiangxi Province, Nanchang, China; ^3^School of Life Sciences, Qufu Normal University, Qufu, China; ^4^State Key Laboratory of Crop Biology, College of Life Sciences, Shandong Agricultural University, Tai’an, China

**Keywords:** *Apis mellifera*, queen, sperm, storage, spermatheca, metabolites, LC–MS, lipids

## Abstract

The polyandrous mating system of honeybees (*Apis mellifera* L.) has garnered widespread attention. Long-lived honeybee queens only mate early in maturation, and the sperm obtained from the aerial mating is stored in the spermatheca. The maintenance of sperm viability in the spermatheca is an intriguing and complex process. However, the key physiological and biochemical adaptations underlying the long-term storage of sperm remain unclear. Analysis of the metabolite profile could help better understand the biology of the spermatheca and offer insights into the breeding and conservation of honeybees and even pest control strategies. Here, the changes in metabolites in the spermatheca were quantified between virgin queens and new-laying queens (with stored sperm) via liquid chromatography–mass spectrometry. Compared with virgin queens, changes occurred in lipids and lipid-like molecules, including fatty acyls and glycerophospholipids (GPL), prenol lipids, and sterol lipids, during storage of sperm in new-laying honeybee queens. Furthermore, the metabolic pathways that were enriched with the differentially expressed metabolites were identified and included GPL metabolism, biosynthesis of amino acids, and the mTOR signaling pathway. The likely roles of the pathways in the maintenance and protection of sperm are discussed. The study identifies key metabolites and pathways in the complex interplay of substances that contribute to the long-term storage of sperm and ultimately reproductive success of honeybee queens.

## Introduction

The honeybee (*A. mellifera* L.) is an important member of the eusocial Hymenoptera, which also includes ants and wasps, and is widely considered to be a paragon of harmonious sociality. Honeybees provide impressive examples of sperm storage. Honeybee queens store sperm in the spermatheca, and therefore, the study of its structure and contents (metabolites) may help to understand the importance of the spermatheca in the long-term storage of sperm. Because queens obtain their lifetime supply of sperm in a single mating flight, which is never replenished after they start laying eggs (Baer et al., 2006), the reproductive success of queens is ultimately sperm-limited (King et al., 2011).

A mature virgin queen typically mates with 12 or more drones during the nuptial mating flight(s) (Seeley, 2010; Simone-Finstrom and Tarpy, 2018). The sperm are transferred into a specialized, spherical organ, the spermatheca, before being used to fertilize eggs. In ways that remain mysterious, viable sperm is preserved throughout the life of a queen (Boomsma et al., 2005). Studies show that female insects interfere with the metabolic rate of stored sperm by reducing the production of harmful reactive oxygen species (ROS) (Reinhardt and Ribou, 2013; Dávila et al., 2015). Therefore, how well a queen has mated may significantly affect the quality of the queen and ultimately the colony health.

The key variables are the number and viability of sperm stored in the spermatheca. Tarpy et al. (2012) measured 80 commercially produced queens and found that queens averaged 4.37 ± 1.45 million stored sperm in the spermatheca with an average viability of 83.70 ± 13.33% (Tarpy et al., 2012). Moreover, queens store only approximately 3–5% of the sperm acquired during copulations (Baer, 2005). Similarly, Oldroyd et al. (1998) found that 5–10% of the sperm of each mate actively migrates to the spermatheca of a queen (Oldroyd et al., 1998). In addition, queens do not exhibit strong behavioral control over the mating number (Tarpy and Page, 2000). Baer et al. (2016) found that queens are remarkably efficient and use only a median of two sperm per egg fertilization with sperm use decreasing in older queens (Baer et al., 2016). Therefore, maintaining viable sperm and using them efficiently are critical in honeybee reproduction.

The female spermatheca receives, maintains, and releases sperm to fertilize eggs and provides an appropriate environment that ensures the long-term viability of sperm (Pascini and Martins, 2017). The reproductive quality of queen bees is central to colony productivity. Despite the remarkable benefit of polyandry to genetic heterogeneity, sperm competition occurs when the sperm of two or more males compete to fertilize the ova of a female (Werner and Simmons, 2008). The seminal fluid of a polyandrous species has a greater positive effect on the survival of a male’s own sperm than on that of the sperm of other males (den Boer et al., 2010). Male sperm may face a trade-off between competitive ability (fast-swimming sperm) and suitability for storage (long-lived and slow-swimming) (Orr and Zuk, 2012).

Environmental stressors, such as widely used pesticides, strongly influence the reproductive potential as well as the genetic diversity (Williams et al., 2015; Forfert et al., 2017). Exposure to these chemicals can lead to colony-wide health problems, even at sublethal levels (Vidau et al., 2011). For example, exposure to fipronil at 0.1 μg/L impairs drone fertility and has detrimental consequences for the reproductive potential of queens (Kairo et al., 2016). The low viability of sperm is indicative of, or linked to, colony performance (Pettis et al., 2016). Temperature can also be an environmental stressor affecting reproductive potential. For example, during shipment, when queens are exposed to temperature extremes (<8°C and >40°C), the mortality of sperm stored in the spermatheca is 50% or higher (Pettis et al., 2016).

In *Drosophila melanogaster*, the compounds in male ejaculate have a short life span (degrading within 7 h after mating) within the female reproductive system (Wolfner, 2011). Thereafter, it appears that only the glandular secretions of the spermatheca sustain the sperm. In the biochemically complex mixture of glandular secretions and stored sperm in the spermatheca, a series of nutritional and protective functions are performed that maintain sperm viability (den Boer et al., 2009; Pascini and Martins, 2017). The metabolic activity of sperm during storage appears to be very important for its survival (Gonzalez et al., 2018). A recent study found that the sperm in the spermatheca is not stationary, and numerous circular movements are observed with the marble-like pattern of the spermatheca changing over time (Tofilski et al., 2018). Similarly, the spermatozoa of *Aedes aegypti* present a circular array configuration, which is also observed in the spermatheca of *Anastrepha suspensa* (Pascini et al., 2012). Because sperm retains respiratory activity in the spermatheca, it is at risk of the damaging effects of ROS common to many biological processes (Collins et al., 2004). The spermatheca of mated queens have higher levels of antioxidant-related proteins than the spermatheca of virgin queens (Gonzalez et al., 2018). These proteins are also in the seminal fluid of drones (Vladimir et al., 2015). Antioxidative enzymes may help reduce the damage caused by ROS, and catalase, glutathione S-transferase (GST), and superoxide dismutase (SOD) contribute to the ability of a queen to store sperm in the spermatheca for several years without loss of viability (Corona and Robinson, 2006). The expression of catalase, thioredoxin 2, and thioredoxin reductase 1 is two times higher in the spermatheca of mated queens than in the spermatheca of virgin queens (Gonzalez et al., 2018). In addition, the sperm from the spermatheca of older queens move more slowly and differ significantly in the activities of lactate dehydrogenase, citrate synthase, and arginine kinase with glyceraldehyde 3-phosphate dehydrogenase the exception (Al-Lawati et al., 2009). According to Paynter et al. (2017), the glycolytic metabolite glyceraldehyde-3-phosphate (GA3P) is a key substrate for honeybee sperm survival and energy production (Paynter et al., 2017). Otherwise, anaerobic energy metabolism of stored sperm predominates in the hypoxic conditions inside the spermatheca in contrast to ejaculated sperm, in which aerobic metabolism predominates. In an analysis of the proteome, Zareie et al. (2013) report a total of 336 bee-specific proteins in the sperm of *A. mellifera* that differ from those of flies and mammals. Within this subset, a substantial number of proteins are predicted to act in enzyme regulation or in nucleic acid binding and processing, which may contribute to the physiological adaptations necessary for the long-term storage of sperm (Zareie et al., 2013).

For the prolonged survival of stored sperm, their disposition and organization in the spermatheca are likely crucial. Poole (1970) and then Zennouche et al. (2015) used scanning electron microscopy and described the disposition of sperm cells inside the spermatheca. They observed coiled spermatozoa in the periphery but uncoiled spermatozoa in the center of the lumen (Zennouche et al., 2015).

Although queens are commonly studied, few reports characterize the metabonomic support for the long-term storage of sperm (den Boer et al., 2008; Paynter, 2015). The studies focus primarily on the differences in metabolites in the spermatheca of queens before and after mating. However, a direct relationship between metabolites and the mating status of a honeybee queen has not been established in any study.

Thus, the aim of the current study was to examine how metabolites in the spermatheca of a new-laying queen changed compared with those in the spermatheca of a virgin queen. An LC–MS approach was used to explore the metabolite profiles and reveal the secrets of the spermatheca.

## Materials and Methods

### Sample Preparation

All samples used in the experiments originated from the apiary of the Apiculture Institute of Shandong Agricultural University, Tai’an, China, in September 2019. Virgin and new-laying honeybee (*A mellifera* L.) queens were obtained with the new-laying queens naturally mated. Virgin queens were the control group.

The spermatheca, which is a spherical sac that holds spermatozoa in a mated queen, was dissected according to the *BEEBOOK* paper on anatomy and dissection of the honeybee (Carreck et al., 2013) using a binocular dissecting microscope (Nikon SMZ745T, Tokyo, Japan) from tissue chilled by dry ice and then immediately transferred to liquid nitrogen and stored at -80°C. Spermatheca (*n* = 100) of virgin (VQS) and new-laying (NLQS) queens were collected and pooled for metabolite extraction.

### Metabolite Extraction

All chemicals and solvents were analytical or HPLC grade. The metabolites were extracted as previously described (Xiong et al., 2018; Jia et al., 2019). Eight replicates (*n* = 100) were analyzed in each group of queens. Briefly, samples were ground at 60 Hz for 2 min and ultrasonicated at ambient temperature (25–28°C) for 10 min. After centrifuging at 10,000 × *g* at 4°C for 15 min, supernatants were dried using a freeze-concentration centrifugal dryer and then resuspended in methanol and water (1:4, v:v), vortexed for 30 s, incubated at 4°C for 2 min, and centrifuged at 10,000 × *g* at 4°C for 5 min. Finally, the solutions were filtered through 0.22 μm microfilters and transferred to LC vials and stored at −80°C until LC–MS analysis.

### Mass Spectrum Data Calling

An Acquity UHPLC (Waters Corporation, Milford, MA, United States) was coupled to an AB SCIEX 5600 TripleTOF System (AB SCIEX, Framingham, MA, United States) (Q Exactive Orbitrap, Thermo Fisher Technologies, Waltham, MA, United States). The LC-MS conditions were described previously (Xiong et al., 2019).

### Data Filtering and Analysis

The LC–MS raw data were analyzed by progqenesis QI v2.3 software (Waters Corporation, Milford, MA, United States) using the following parameters: precursor tolerance, 5 ppm; fragment tolerance, 10 ppm; retention time (RT) tolerance, 0.02 min; noise elimination level, 10.00; minimum intensity, 15% of base peak intensity. Any peaks with missing values (ion intensity = 0) in more than 50% of samples were removed, further reducing the resulting matrix. An internal standard was used for data quality control (QC). The QC samples were prepared by mixing aliquots of all samples into a pooled sample that was injected at regular intervals (every 10 samples) throughout an analytical run to provide a set of data in which repeatability could be assessed.

Progenesis QI (Waters Corporation, Milford, MA, United States) data processing software identified the metabolites using public databases, such as http://www.hmdb.ca/ and http://www.lipidmaps.org/, and self-built databases (Lu-Ming Biotech Co., Ltd., Shanghai, China).

**FIGURE 1:**
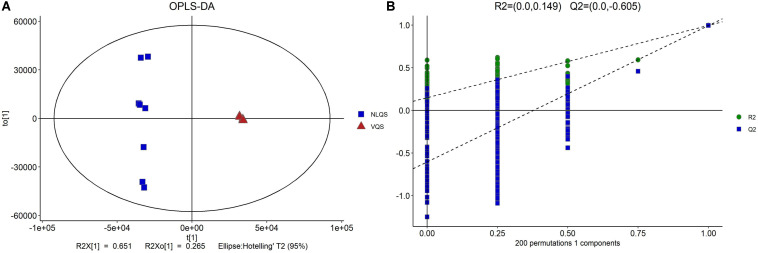
Multivariate statistical analyses of the differences in metabolites between NLQS and VQS in the honeybee *Apis mellifera*. **(A)** Orthogonal partial least-squares-discriminant analysis (OPLS-DA) was conducted using the OmicShare cloud platform powered by Gene *Denovo* Biotechnology Co., Ltd https://www.omicshare.com/tools/. *R*^2^X = 0.651; *R*^2^Xo = 0.265; Ellipse: Hotelling’ T2 (95%). **(B)** Permutation test plot. *R*^2^ = (0.0, 0.149); *Q*^2^ = (0.0, -0.605).

To identify the differentially expressed metabolites, principal component analysis (PCA) and orthogonal partial least-squares-discriminant analysis (OPLS-DA) (Worley and Powers, 2016) of combined positive and negative data of ion modes were conducted, and the metabolite diversities were visualized between the groups. The variable importance in the projection (VIP), a weighted sum of PLS loading that is commonly to used to identify the key characters in metabonomic data (Lin et al., 2011), was used to rank the overall contribution of each variable to the OPLS-DA model, and those variables with VIP > 1 and *p* < 0.05 (two-tailed Student’s *t*-test) were considered as differentially expressed metabolites. In addition, the 7-fold cross-validation and response permutation test (*n* = 200) was adopted to validate the model (Figure 1B). The *R*^2^ and *Q*^2^ validation plot shown in Figure 1B indicates that the model was credible without overfitting.

Pearson’s correlation coefficients were calculated to analyze the correlations between the VIP values of metabolites in NLQS and VQS.

The relative abundance of differentially expressed metabolites was further analyzed by metabolic pathway enrichment with the metabolites mapped to the Kyoto Encyclopedia of Genes and Genomes (KEGG) database (freely available at http://www.kegg.jp/). To identify significantly enriched pathways, the *p-*value of each acquired metabolic pathway was measured using a hypergeometric test (Ortmayr et al., 2019) and false discovery rate (FDR) correction (Xie et al., 2011), and the metabolic pathways with *p* < 0.05 were retained. In addition, compound names were uploaded to pathway analysis via MetaboAnalyst^1^ (Xia and Wishart, 2010). Specific pathway analysis algorithms of the hypergeometric test were used in over-representation analysis and relative-betweenness centrality for pathway topology analysis and in mapping to the pathway library of *Drosophila melanogaster* (fruit fly, KEGG, version: current, October 2019).

## Results

### Metabonomic Changes in the Spermatheca Between Virgin Queens and New-Laying Queens

To determine the overall metabolomic changes in the spermatheca of *A. mellifera* L. after mating, the metabolites in the spermatheca of NLQS were compared with those in the spermatheca of VQS using LC–MS. After filtering the data, the clean data were used in further analyses. PCA and an OPLS-DA score plot (Figure 1A) identified obvious differences in the metabolites between NLQS and VQS.

A total of 7745 metabolites were identified from 19,016 substance peaks (Supplementary Table S1). The lipids and lipid-like substances included fatty acyls and glycerophospholipids (GPL), prenol lipids, and sterol lipids with several specific substances, such as fatty acids and conjugates, fatty esters, and fatty acid esters.

### Analysis of the Main Differential Metabolites

**FIGURE 2:**
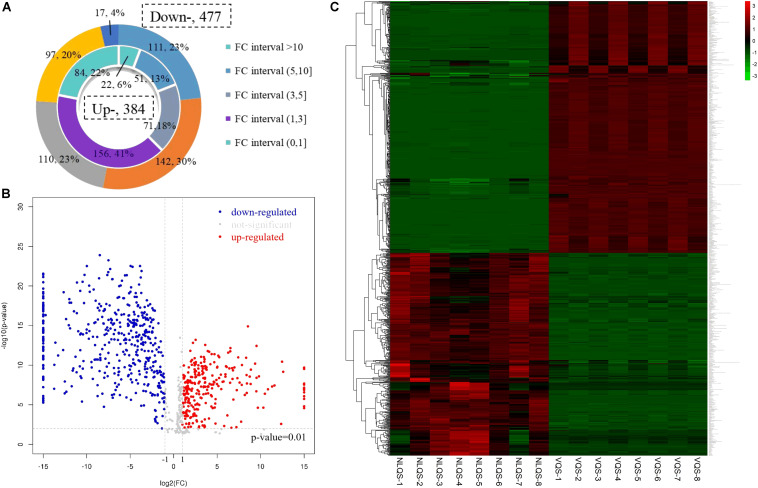
Fold change (FC) arrangement and pattern of metabolites that were different in NLQS compared with those in VQS in the honeybee *Apis mellifera*. **(A)** The inner circle indicates high concentration metabolites, and the outer circle indicates low concentration metabolites in NLQS compared with those in VQS. **(B)** Volcano plot of metabolites that were different in NLQS compared with those in VQS. Each dot represents an individual metabolite: red indicates high concentration, blue indicates low concentration, and gray indicates no change. **(C)** Pattern heat map. The color code from green to red indicates the abundance of metabolites from low to high, respectively.

A total of 861 metabolites were obtained via LC–MS that met the VIP threshold (VIP > 1) of the OPLS-DA model and had a significant *t*-test (*p* < 0.05) (Li et al., 2019). Compared with VQS, 384 (44.60%) metabolites were of high and 477 (55.40%) were of low concentration in NLQS (Figure 2A). Further analyses are shown in Figure 2B, indicating that the mating status (mated or virgin queen) led to differences in the metabolites. As shown in Figure 2C, the total pattern of the metabolites was distinct between VQS and NLQS with consistency among biological replicates. Therefore, the 861 differentially expressed metabolites were screened in abundance (fold change) and weight (VIP) analyses (Table 1 and Supplementary Table S2).

**TABLE 1:** Top 50 metabolites that were qualitatively significantly different in NLQS compared with those in VQS of honeybee *Apis mellifera.*

RT (min)	Ion mode	Metabolites	VIP	*P*-value	adj.*P*-value	log2(FC)
0.71	pos	Glycerophosphocholine	28.29	0.00	0.00	2.97
11.26	pos	PC(O-18:1(11Z)/0:0)	24.12	0.01	0.01	6.63
8.73	pos	PC(18:3(9Z,12Z,15Z)/0:0)	23.66	0.00	0.00	1.13
0.68	pos	Pyro-L-glutaminyl-L-glutamine	22.88	0.00	0.00	2.82
13.55	pos	PC[18:2(9Z,12Z)/18:4(6Z,9Z,12Z,15Z)]	16.74	0.00	0.00	2.41
0.69	pos	3-Hydroxychavicol 1-[rhamnosyl-(1- > 6)-glucoside]	15.80	0.00	0.00	9.81
9.86	pos	PC(18:1(6Z)/0:0)	14.51	0.00	0.00	0.74
0.69	pos	Phosphocholine	14.01	0.00	0.00	5.13
8.73	neg	LysoPC[18:3(6Z,9Z,12Z)]	13.79	0.00	0.00	1.26
0.66	pos	Isoswertisin 2^″^-O-(2′″-methylbutyrate)	13.12	0.00	0.00	8.42
10.10	neg	LysoPC[18:1(9Z)]	12.55	0.00	0.00	0.65
15.25	pos	PC[18:1(11Z)/18:3(6Z,9Z,12Z)]	12.36	0.00	0.00	0.57
1.12	pos	L-Isoleucine	12.26	0.00	0.00	1.76
16.39	pos	PC[18:1(9Z)/16:1(9Z)]	11.86	0.00	0.00	0.96
1.60	pos	trans-Cinnamic acid	10.06	0.00	0.00	1.50
9.87	neg	PC(18:1(11Z)/0:0)	9.93	0.00	0.00	0.96
10.99	neg	LysoPC(18:0)	9.77	0.00	0.00	1.31
8.69	pos	LysoPE(18:3(9Z,12Z,15Z)/0:0)	9.46	0.00	0.00	0.80
10.24	pos	PI(18:1(9Z)/0:0)	9.39	0.00	0.00	–2.08
10.96	neg	LysoPE(18:0/0:0)	9.33	0.02	0.02	0.75
8.97	neg	LysoPC(16:1(9Z)/0:0)	9.15	0.00	0.00	1.47
11.28	neg	LysoPC(P-18:0)	8.90	0.01	0.01	7.21
11.22	neg	L-a-Lysophosphatidylserine	8.56	0.02	0.02	1.43
11.32	pos	PC(O-18:0/0:0)	8.42	0.03	0.04	4.78
0.69	pos	L-2-Amino-5-hydroxypentanoic acid	8.37	0.00	0.00	0.99
11.25	pos	13R-HODE	8.15	0.00	0.00	2.56
12.12	neg	1-Stearoylglycerophosphoinositol	8.12	0.02	0.02	1.45
10.74	pos	enantio-PAF C-16	8.06	0.01	0.01	1.97
0.69	pos	3,4,5-trihydroxy-6-{[5-(4-methoxyphenyl)-3-oxopentan-2-yl]oxy}oxane-2-carboxylic acid	7.77	0.00	0.00	12.46
10.51	pos	MG(0:0/18:3(9Z,12Z,15Z)/0:0)	7.45	0.00	0.00	6.99
10.04	neg	LysoPE(18:1(11Z)/0:0)	7.42	0.02	0.02	0.39
14.08	pos	PC(18:4(6Z,9Z,12Z,15Z)/16:0)	7.28	0.00	0.00	2.20
16.36	pos	PE[15:0/20:1(11Z)]	7.12	0.00	0.00	1.29
8.55	neg	LysoPC[18:3(9Z,12Z,15Z)]	6.97	0.00	0.00	1.84
2.76	neg	FAD	6.96	0.00	0.00	3.55
6.09	pos	(5-butyl-6-methyloctahydroindolizin-8-yl)methanol	6.95	0.00	0.00	–8.42
14.21	pos	PC[18:2(9Z,12Z)/18:3(9Z,12Z,15Z)]	6.21	0.00	0.00	1.70
9.81	neg	LysoPC(0:0/16:0)	6.07	0.02	0.03	0.82
0.94	pos	arabinofuranosylguanine	6.02	0.00	0.00	1.12
1.09	pos	N-(1-Deoxy-1-fructosyl)leucine	5.95	0.00	0.00	3.62
2.61	pos	Methoxyzotepine	5.90	0.00	0.00	3.41
10.71	pos	LysoPE(0:0/18:0)	5.87	0.04	0.05	1.11
0.94	neg	(S)-2-Hydroxyglutarate	5.85	0.00	0.00	4.91
9.83	pos	LysoPE[0:0/18:1(11Z)]	5.82	0.01	0.01	0.50
12.06	pos	PI(18:0/0:0)	5.77	0.03	0.03	1.34
4.85	pos	N-methylundec-10-enamide	5.75	0.00	0.00	–8.45
8.85	neg	PI(16:1(9Z)/0:0)	5.73	0.00	0.00	–2.44
16.08	neg	N’-nitrosoanabasine	5.69	0.00	0.00	0.60
15.24	pos	PC[16:0/18:3(6Z,9Z,12Z)]	5.65	0.00	0.00	0.72
15.56	pos	SM(d18:1/16:0)	5.63	0.00	0.00	–0.44

*RT (min) represent retention time. pos/neg means the metabolite profiling was obtained via ESI positive or ESI negative ion modes.*

**FIGURE 3:**
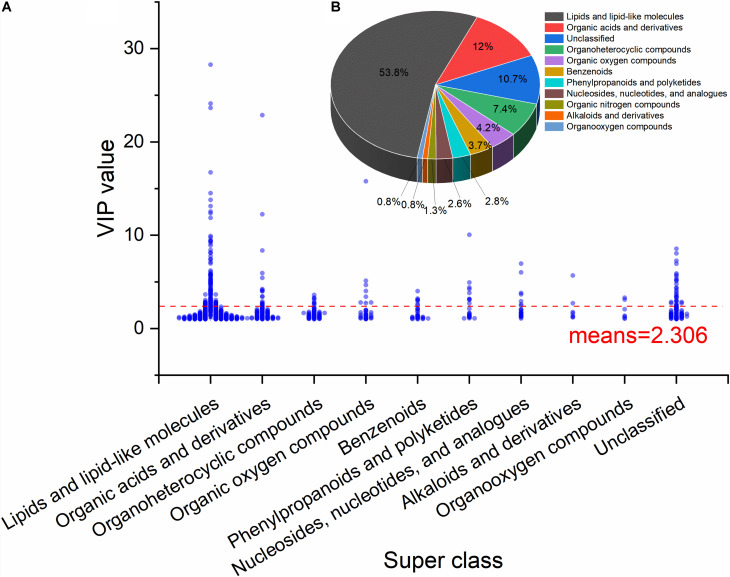
Distribution of variable importance in the projection (VIP) values and classification of the differentially expressed metabolites in the comparison of NLQS with VQS in the honeybee *Apis mellifera*. **(A)** Scatter plot of the VIP distribution in each superclass of metabolite. The red dashed line represents the population mean value (2.306) of the differentially expressed metabolites. **(B)** Pie chart of the proportion of each superclass of metabolite. The superclass of lipids and lipid-like molecules accounted for 53.8%, followed by organic acids and derivatives at 12.0% and unclassified at 10.7%.

Figure 3A shows the VIP values of the metabolites classified according to superclass. During storage (NLQS), the category of lipids and lipid-like molecules had the highest weight. The compound glycerophosphocholine (GPC) had a VIP value of 28.29 although the mean was 2.306. As shown in Figure 3B, the most abundant differentially expressed metabolites were lipids and lipid-like molecules, which accounted for 53% of the total, followed by organic acids and derivatives at 12%.

**FIGURE 4:**
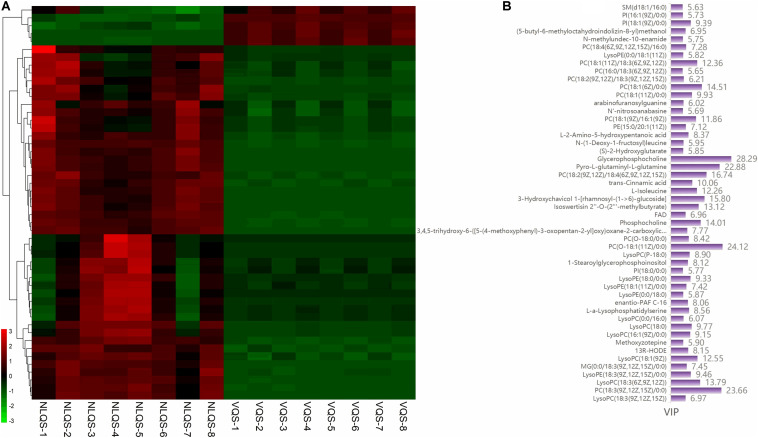
Heat map of the hierarchical clustering analysis of the top 50 differentially expressed metabolites in the comparison of NLQS with VQS in the honeybee *Apis mellifera*. **(A)** Differentially expressed metabolites were separated using hierarchical clustering. The x-axis has the eight (1–8) biological replicates of each type of spermatheca, and the y-axis represents the differentially expressed metabolites separated using hierarchical clustering. The color from green to red indicates an increase in abundance of metabolites from low to high, respectively. **(B)** VIP values of each differentially expressed metabolite.

**FIGURE 5:**
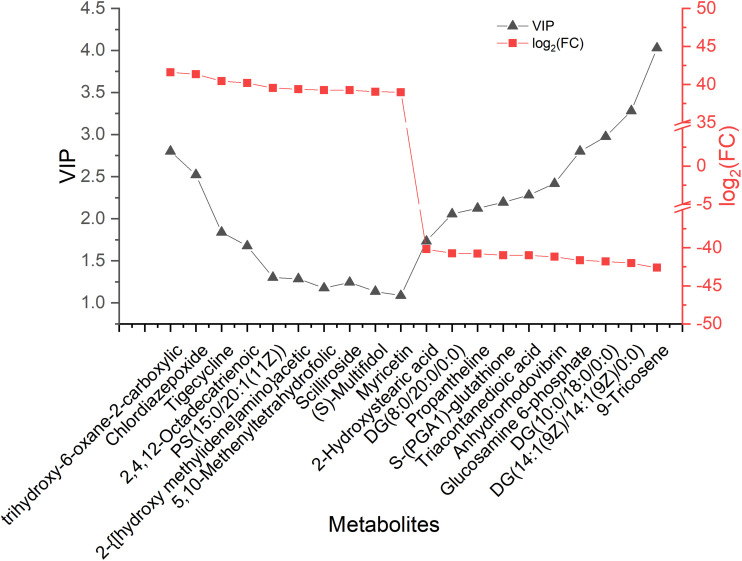
Point diagram of 20 differentially expressed metabolites in the comparison of NLQS with VQS in the honeybee *Apis mellifera*. The gray points represent variable importance in the projection (VIP) values, and red points represent log_2_ (fold change, FC) values. The metabolites in the left half were high, whereas those in the right half were low concentration in new-laying queen spermatheca compared with those in virgin queen spermatheca.

To show the relative contents of the significantly differentially abundant metabolites, hierarchical cluster analysis (HCA) was performed. The HCA heat map is shown Figure 4A, and the histogram of the top 50 metabolites is shown in Figure 4B. The levels of most of the altered metabolites were more abundant in NLQS compared with those in VQS, suggesting that long-term storage led to stronger fluctuations in the metabolites. Notably, GPC, pyro-L-glutaminyl-L-glutamine, and L-isoleucine increased significantly in NLQS, and the larger VIP values indicated a greater contribution during storage. The 20 most differentially expressed metabolites are shown in Figure 5 and Supplementary Table S3.

**FIGURE 6:**
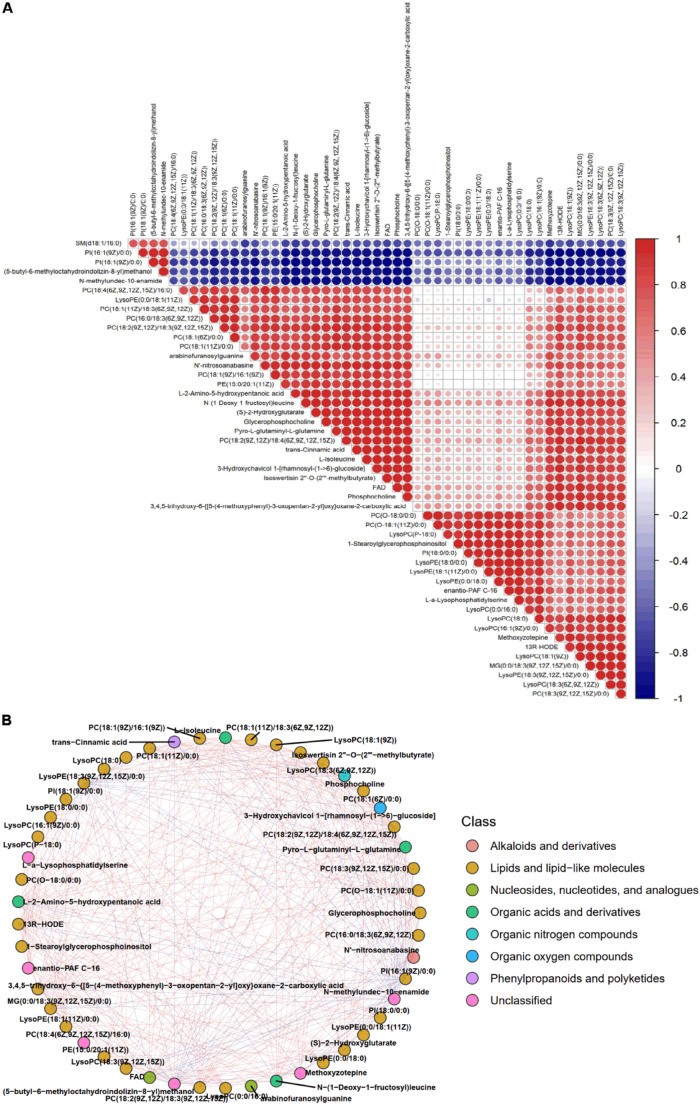
Correlation analysis and network of differentially expressed metabolites in the comparison of NLQS with VQS in the honeybee *Apis mellifera*. **(A)** Pearson’s coefficients of correlation of variable importance in the projection values were used to analyze the correlations among metabolites between NLQS and VQS. Red, positive correlation; blue, negative correlation. Different sizes of circles indicated the correlation of Pearson’s coefficients. **(B)** Network of interactions among classes of differentially expressed metabolites. The Pearson correlation coefficient threshold was set to 0.9. Red lines represent positive correlations between substances, and blue lines represent negative correlations between substances.

Correlation analysis was used to measure the correlations between VQS and NLQS metabolites with significant differences in order to further understand the relationships between metabolites in the process of storage. Figure 6A shows the Pearson correlation coefficients of the correlations of VIP values of metabolites in VQS and NLQS. The network in Figure 6B and Supplementary Table S4 present the interactions among metabolites with correlation coefficients greater than 0.9.

### Metabolic Pathways Affected by Changes in Metabolites

**FIGURE 7:**
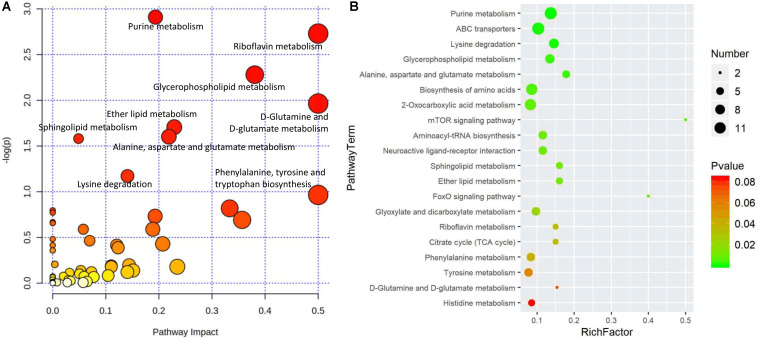
Pathway enrichment analysis of the differentially expressed metabolites in the comparison of NLQS with VQS in the honeybee *Apis mellifera*. **(A)** Pathway impact analysis using the joint pathway analysis module (Chong et al., 2019). The color from white to red represents the larger *p*-value, and the size of each node corresponds to its pathway impact value from the pathway topology analysis, respectively. **(B)** Heat map analysis of the metabolic pathways enriched by the differentially expressed metabolites. The analysis was based on a visualization analysis of metabolic pathways (Xia and Wishart, 2010) originated from KEGG http://www.kegg.jp/. From green to red indicates increasing *p-*values; the point size indicates the number of metabolites enriched in each pathway.

To further analyze the biochemical changes, pathway enrichment analysis was conducted based on the KEGG pathways database. The metabolic pathways that were significantly enriched with the differentially expressed metabolites were involved in lipid metabolism, purine metabolism, ABC transporters, and amino acid metabolism. Figure 7 shows the top 20 metabolic pathways that were enriched by the differentially expressed metabolites.

According to the enrichment analysis, 17 of 72 pathways (purine metabolism, ABC transporters, lysine degradation, and GPL metabolism, among others) were significantly enriched with *p*-values less than 0.05 (Table 2 and Supplementary Table S5). The key metabolites that increased included those associated with GPL metabolism, sphingolipid metabolism, and the mTOR-signaling pathway, and those that decreased were associated with the tricarboxylic acid (TCA) cycle. These processes are essential components of lipid, amino acid, and carbohydrate metabolic pathways to gain energy and support cell metabolism.

**TABLE 2:** Metabolic pathway enrichment analysis based on *p*-value no more than 0.05.

#	ID annotation	Annotation	Match status	RichFactor	*p*-value	-lg(*p*-value)	FDR correction
1	ame00230	Purine metabolism	13/95	0.136842105	2.1276E-05	4.672109906	0.000776574*
2	ame02010	ABC transporters	13/126	0.103174603	0.000410312	3.386885643	0.009984262*
3	ame00310	Lysine degradation	8/55	0.145454545	0.000590325	3.22890873	0.010773434*
4	ame00564	Glycerophospholipid metabolism	7/52	0.134615385	0.002088801	2.680102851	0.0304965*
5	ame00250	Alanine, aspartate and glutamate metabolism	5/28	0.178571429	0.002623066	2.581190834	0.031913966*
6	ame01230	Biosynthesis of amino acids	11/128	0.0859375	0.005034777	2.298019803	0.052505526
7	ame01210	2-Oxocarboxylic acid metabolism	11/134	0.082089552	0.007107752	2.148267725	0.057864678
8	ame04150	mTOR signaling pathway	2/4	0.5	0.007134001	2.146666812	0.057864678
9	ame00970	Aminoacyl-tRNA biosynthesis	6/52	0.115384615	0.009365324	2.028477182	0.059827478
10	ame04080	Neuroactive ligand-receptor interaction	6/52	0.115384615	0.009365324	2.028477182	0.059827478
11	ame00600	Sphingolipid metabolism	4/25	0.16	0.010654208	1.97247881	0.059827478
12	ame00565	Ether lipid metabolism	4/25	0.16	0.010654208	1.97247881	0.059827478
13	ame04068	FoxO signaling pathway	2/5	0.4	0.01161524	1.93497183	0.060565178
14	ame00630	Glyoxylate and dicarboxylate metabolism	6/62	0.096774194	0.021249264	1.672656108	0.103413085
15	ame00740	Riboflavin metabolism	3/20	0.15	0.031869127	1.496629829	0.136849782
16	ame00020	Citrate cycle (TCA cycle)	3/20	0.15	0.031869127	1.496629829	0.136849782
17	ame00360	Phenylalanine metabolism	6/72	0.083333333	0.040654726	1.390888959	0.164877501

Match Status = Hit/Total. The Total is the total number of compounds in the pathway; the Hits is the actually matched number. The p-value is the originally calculated from the enrichment analysis; the −lg(p-value) is the p-value adjusted by logarithm operation based on 10; the FDR is the p value adjusted using False Discovery Rate results from the pathway analysis. *FDR < 0.05.

## Discussion

The spermatheca of a female can substantially extend the life span and functionality of sperm during storage (Orr and Zuk, 2012). The understanding of the long-term storage of sperm in honeybee queens is enriched by decades of studies involving morphology (Dallai, 1975), biochemistry (Al-Lawati et al., 2009), and multiomics analysis (Zareie et al., 2013). Much has been learned about the commonly studied molecular mechanisms of oxidation and ROS. By contrast, the metabolites that support the long-term storage of sperm have not been well documented. In stored insect sperm, the metabolic rate and the production of oxygen radicals are suppressed (Ribou and Reinhardt, 2012). Recent advances in the identification and quantification of metabolites have increased understanding of the complex process, and metabolites are implicated in various biological cascades through the regulation of phosphorylation, acetylation, and peroxidation (Baer and Millar, 2016). Lipids, organic acids, and organoheterocyclic compounds involved in lipid and amino acid metabolism may play important roles in the storage.

### Changes in Lipid Metabolism in New-Laying Queen Spermatheca vs. Virgin Queen Spermatheca

**FIGURE 8:**
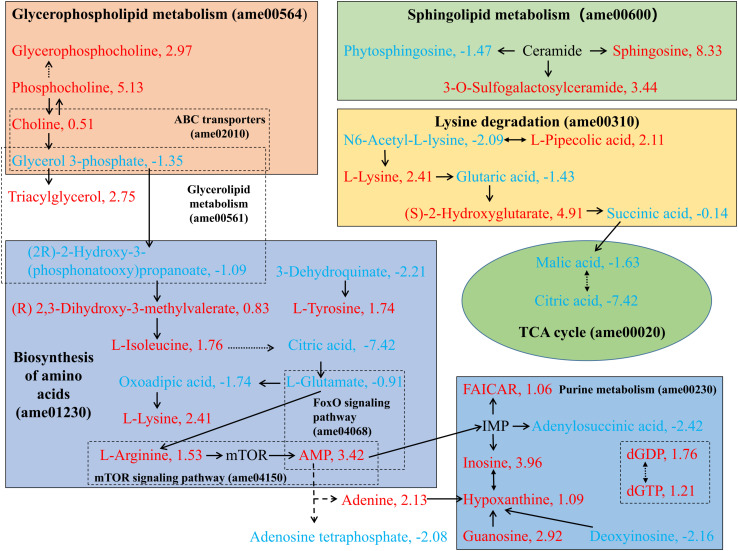
Summary of the primary metabolic pathways that changed in the comparison of NLQS with VQS in the honeybee *Apis mellifera*. The pathways were summarized and outlined based on the KEGG database (see footnote). The profiles of differentially expressed metabolites were obtained from the LC–MS of metabolomic data. The text color indicates the level of change in the NLQS compared with that in the VQS: a red, positive number indicates a higher level; a blue, negative number indicates a lower level. Solid arrows indicate direct action; dashed arrows indicate a remote-action relationship; and double-sided arrows indicate interaction.

Lipid metabolism has an important role in the aging process (Johnson and Stolzing, 2019). (2R)-2-hydroxy-3-(phosphonatooxy) propanoate (C00197), known as glycerate-3p, is a key substrate for sperm survival and energy production (Paynter, 2015). Owing to the anaerobic environment, the content of glycerate-3p decreased after storage in the spermatheca (Figure 8). In addition, the concentration of 5,10-methenyltetrahydrofolic acid and mesaconic acid was significantly different between NLQS and VQS with the log_2_(FC) values of 39.26 and −39.34, respectively. In addition, citric acid was far less than in NLQS compared with that in VQS with the log_2_(FC) value of −7.42 (Supplementary Table S2). In *Drosophila Sply*^05091^ mutants (lack of functional sphingosine-1-phosphate lyase), accumulated sphingolipids trigger elevated levels of apoptosis in reproductive organs and lead to supernumerary spermatheca and degenerated ovaries via the modulation of sphingolipid signaling pathways (Phan et al., 2007).

Membrane integrity is vital to maintain sperm viability and motility, which are essential for fertilization (Gonzalo et al., 2018). Sperm are highly susceptible to oxidative stress because ROS and reactive nitrogen species target cellular membranes (Aitken, 2017). Mixtures with GPL can ameliorate oxidative stress in cells (Gonzalo et al., 2018), and indeed, sperm phospholipids are protected from oxidation during storage (Wegener et al., 2013).

In this study, 153 of 861 metabolites were in the class GPL, under the superclass of lipids and lipid-like molecules, which was the largest classification. The abundant lipids provide assistance to the sperm against peroxidation via superoxide dismutase and the glutathione peroxidase/reductase system (Gonzalo et al., 2018). A total of 68 GPCs and 41 glycerophospho-ethanolamines (GPEs) were obtained, which were the most abundant metabolites regardless of whether the putative compounds were prefixed with lysopc/pc or lysope/pe. Wegener et al. (2013) report that the lipids in drone sperm are dominated by two GPC species with small amounts of sphingomyelins and GPEs also detected. They found the composition of sperm lipids does not change even when stored for years (Wegener et al., 2013).

The low level of oxygen inside the spermatheca tends to favor anaerobic metabolism (Agarwal et al., 2003). Therefore, the stored sperm in the NLQS was challenged by low energy production as well as the need to avoid ROS damage. The phospholipids of the sperm plasma membrane have a high content of polyunsaturated fatty acids represented in their acyl moieties (Storey, 2008). In further analysis of the class fatty acids, 72 of 86 metabolites were of light concentration with lower VIP values in NLQS than in VQS (Supplementary Table S6), indicating that the concentrations of fatty acids decreased after queen mating. The protective mechanisms operating inside the spermatheca were presumed to act via fatty acid peroxidation. Generally, lipids are among the molecules most affected by uncontrolled oxidation (Wegener et al., 2013).

### Amino Acid and Polyamine Acid Metabolism

Among the compounds in the lysine degradation pathway (ame00310), the decreases in concentrations of N6-acetyl-L-lysine, glutaric acid, and succinic acid in stored sperm might have led to reductions in downstream pathway activity. The TCA cycle pathway (ame00020) is a major enzyme-catalyzed cascade that forms a key part of aerobic respiration in cells (du Plessis et al., 2015). It may seem surprising that few ATPs are produced directly by the TCA cycle, much less than by glycolysis and oxidative phosphorylation (Plessis et al., 2015). Because of the high probability of anaerobic respiration, malic acid and citric acid were of low concentration in NLQS (Figure 8).

The biosynthesis of amino acids (ame01230) consisted of an integrated network of pathway modules that included carbohydrate and amino acid metabolism. Amino acids, such as L-glutamine (at 20 mM) and L-proline (at 25 mM), are also used as semen additives in sperm cryopreservation because they prevent cryoinjuries to sperm and improve the prefreeze and postthaw semen characteristics (Sangeeta et al., 2015). The content of L-glutamine was 7.04 times higher in NLQS than that in VGS, which is similar to L-isoleucine (3.39-fold) (Supplementary Table S7).

Amino acids play multiple roles in various metabolism. According to Pratavieira et al. (2020), the execution of the proboscis extension response requires the metabolic transformations of arginine, ornithine, and lysine as substrates for the production of putrescine, cadaverine, spermine, spermidine, 1,3-diaminopropane, and γ-aminobutyric acid (Pratavieira et al., 2020). The metabolite L-arginine had a higher level of fold change (2.90 times) in NLQS than in VQS with the VIP of 5.44, similar to L-lysine with fold change of 5.30 and VIP of 2.52.

### Other Features of Metabolic Change

Acetylation could be involved in the acquisition of fertilization competence in mammalian sperm (Ritagliati et al., 2018). In acetylproteome studies with human sperm, both PKAc and PKARII are acetylated during capacitation (Guohai et al., 2014; Yu et al., 2015). In the present work, a downward trend in acetylation was observed in sperm stored in the spermatheca (Supplementary Table S8). After transfer into the spermatheca, stored sperm is generally assumed to be inactive until activated during fertilization by Zrt- and Irt-like protein transporter 7.1 (ZIPT-7.1) (Zhao et al., 2018) (not detected in the present study) and mineral zinc (Chu, 2018).

Sakaguchi et al. (2009) studied bovine sperm–oocyte interactions and discovered that supplementary N-acetyl-D-glucosamine (GlcNAc) (at 5 or 25 mM) significantly suppresses the number of sperm that attach to the zona pellucida (Sakaguchi et al., 2009). Moreover, a recommended concentration of 0.05% soluble GlcNAc significantly increases the cryopreservation of boar spermatozoa (Yi et al., 2002). In this study, GlcNAc and glucosamine 6-phosphate (GlcNAc-6P) in amino sugar and nucleotide sugar metabolism (ame00520) had significantly lower contents in NLQS than in VQS with log_2_(FC) values of −34.96 and −41.65, respectively.

## Data Availability Statement

The original contributions presented in the study are included in the article/Supplementary Material, further inquiries can be directed to the corresponding author.

## Author Contributions

ZL, BX, FL, and XG conceived and designed the experiments. ZL and FL performed the experiments. ZL, YW, HW, and GZ analyzed the data. LM and KH conducted the breeding of animal material. ZL, FL, and BX contributed reagents, materials and analysis tools. ZL and GL wrote the manuscript. All authors contributed to the article and approved the submitted version.

## Conflict of Interest

The authors declare that the research was conducted in the absence of any commercial or financial relationships that could be construed as a potential conflict of interest.

## Footnotes

1 https://www.metaboanalyst.ca/

## Supplementary Material

Supplementary Table 1 Statistics of the number of peaks.

Supplementary Table 2 Differentially expressed metabolites in NLQS compared with VQS.

Supplementary Table 3 Top 20 metabolites based on fold change that showed major changes in NLQS compared with VQS.

Supplementary Table 4 Pearson’s coefficients of correlation between metabolites in NLQS and VQS.

Supplementary Table 5 Metabolic pathway enrichment analysis.

Supplementary Table 6 Metabolites in the class fatty acids.

Supplementary Table 7 Essential amino acid metabolites.

Supplementary Table 8 Acetyl-related metabolites.
