# Supplementary material for: Metabolite Support of Long-Term Storage of Sperm in the Spermatheca of Honeybee (Apis mellifera) Queens
Source: Front Physiol. 2020 Nov 10;11:574856. doi: 10.3389/fphys.2020.574856 (PMC7683436; doi:10.3389/fphys.2020.574856)
Supplement: Supplementary Table 1 — Statistics of the number of peaks. [file Table_1.DOCX]

**Table S1.** Statistics of the number of peaks

| Category | All | Negative-ion | Positive-ion |
| --- | --- | --- | --- |
| substance peaks | 19016 | 4275 | 14741 |
| metabolites | 7745 | 1264 | 6481 |

Footnote: The metabolic profiling was obtained both in both ESI positive and ESI negative ion modes using ACQUITY UPLC I-Class system (Waters Corporation, Milford, USA) coupled with Vion IMS Q-Tof Mass spectrometer( Waters Corporation, Milford, USA)
